# Microbial Removals by a Novel Biofilter Water Treatment System

**DOI:** 10.4269/ajtmh.14-0001

**Published:** 2015-04-01

**Authors:** Christopher Wendt, Rebecca Ives, Anne L. Hoyt, Ken E. Conrad, Stephanie Longstaff, Roy W. Kuennen, Joan B. Rose

**Affiliations:** Water Quality, Environmental, and Molecular Microbiology Laboratory, Department of Fisheries and Wildlife, Michigan State University, East Lansing, Michigan; Amway, Ada, Michigan

## Abstract

Two point-of-use drinking water treatment systems designed using a carbon filter and foam material as a possible alternative to traditional biosand systems were evaluated for removal of bacteria, protozoa, and viruses. Two configurations were tested: the foam material was positioned vertically around the carbon filter in the sleeve unit or horizontally in the disk unit. The filtration systems were challenged with *Cryptosporidium parvum, Raoultella terrigena*, and bacteriophages P22 and MS2 before and after biofilm development to determine average log reduction (ALR) for each organism and the role of the biofilm. There was no significant difference in performance between the two designs, and both designs showed significant levels of removal (at least 4 log_10_ reduction in viruses, 6 log_10_ for protozoa, and 8 log_10_ for bacteria). Removal levels meet or exceeded Environmental Protection Agency (EPA) standards for microbial purifiers. Exploratory test results suggested that mature biofilm formation contributed 1–2 log_10_ reductions. Future work is recommended to determine field viability.

## Introduction

An estimated 884 million people currently rely on unimproved drinking water sources including unprotected wells and springs; small and large water tanks; surface water (e.g., rivers, dams, canals, and irrigation channels).^1^ The majority of people without access to clean water and/or sanitation (more than 70%) reside in rural areas.^1^ Unsafe drinking water, lack of sanitation, and poor hygiene rank fourth in global health risk factors as measured in disability-adjusted life years (DALYs).^2^ According to the United Nations Organization for Education, Science and Culture (UNESCO) this problem contributes to approximately 3.5 million deaths each year.^3^ This risk is increased among low-income populations worldwide, particularly in sub-Saharan Africa and southeast Asia.^2^

Often in these areas, access to clean water requires long trips to a distant source, usually on foot. This transportation process can increase the risk of contamination.^2^ A link between the distance walked for clean water and negative health outcomes for children in particular has been identified.^4^ Point-of-use devices, especially those that are inexpensive and do not require electricity, have shown promise for providing clean potable water to at-risk populations, diminishing the problems associated with water quality.^5^ The systems tested in this study could potentially fill this role to improve the drinking water situation of these areas.

The usefulness of a point-of-use device cannot be measured strictly by the reduction of pathogenic organisms, a complete view of a device must be considered before deployment. Biological, economic, and behavioral factors all play a role in determining devices ultimate effectiveness in the field. A filtration system must be able to maintain adequate levels of microbial reduction for long periods as well as be able to process large volumes of water.^6^ A focus on simplicity would serve designers well in producing a useful point-of-use device. A device that is simple to set up, simple to use, and straightforward to maintain will stand the best chance to see extended use in rural environments. The filtration process must not add considerable labor time to a household lest they abandon it for more productive activities.^6^ Another consideration must be transport, a device that is difficult or expensive to transport will not be worth deploying regardless of how effective it may be in the field. Cost is another major factor in long-term compliance; not only must the system be inexpensive initially but maintenance costs should also be low to keep the system running properly without service interruption.^6^ Finally, the supply of supplemental substances such as chlorine and other chemicals and other parts must be available to keep service running.^6^

Gravity-fed point-of-use devices, specifically, have been proven effective in mitigating gastrointestinal disease in many of these scenarios.^6^ These designs have been shown to be durable and effective for several years^7^ and include multilayered sand filters referred to as “biosand” systems that rely on removal of pathogens rather than inactivation of microorganisms, contributing to their sustainability. The biosand design has demonstrated the ability to significantly reduce the number of bacteria, some viruses, and parasites in laboratory and field studies.^8^–^10^ Biosand filters have been used successfully in rural communities, showing greater than 85% continued use within households for up to 8 years after implementation.^6^ Two studies using the biosand filter system, one in the River Njoro watershed in Kenya and one in the Dominican Republic, demonstrated significant reduction in days the population experienced diarrhea (specifically children aged 0–15 years in the Kenyan study).^9^,^10^

The biosand design does suffer from some drawbacks. It can be time consuming to deploy, the sand/gravel component makes it heavy and/or costly to transport, or reliant on local materials for the media. Maintenance of the biosand system can also be cumbersome if it requires a complete change of all the internal sand and gravel.

Incorporated as part of the design of some point-of-use filters are layers of organic material and benign growth of microorganisms into a structure known as biofilms. Biofilms have been shown to capture and retain a range of microbes from water, particularly protozoa^8^,^11^,^12^ as well as remove organic matter that contributes to foul tastes and smells.^13^–^15^ It also allows for the possibility of predation of pathogens by other organisms within the biofilm.^16^ Growth of the biofilm layer is a result of the natural use of the system. Development of biofilms in designated areas of the system allows for these possible mechanisms of removal with minimal effort and no additional cost.

The objective of this research study was to evaluate the removal of select microorganisms by two novel point-of-use filtration designs. These “biofilter” systems, as they will be referred to in this article, use a foam material to support the growth of biofilms. The biofilter designs are intended as possible alternatives to other gravity-fed point-of-use filters such as the biosand system. They are intended as potential new candidates for deployment among rural populations requiring in-home point-of-use water filtration devices. The biofilter systems have no sand or gravel layers, which could make transport, deployment, and maintenance easier in some respects. The biofilter gravity-fed system uses a multiple barrier approach including water biofiltration and a delivered chlorine disinfection. A multi-barrier approach has been shown to be effective in producing a robust process capable of removing and/or inactivating a variety of microbial pathogens.^17^ This research study did not compare the biofilter design to the biosand design directly. The first and most important test for a new filtration system is its ability to remove and reduce the concentration of pathogens within the influent waters. Only after this basic question is answered, should more peripheral considerations such as practicality, cost, and performance under extenuating circumstances be addressed. This research study is intended to answer this basic question of performance and briefly explore some of the other concerns.

## Materials and Methods

Two multi-barrier point-of-use drinking water treatment systems using a carbon filter and foam material were compared for removal of bacteria, protozoa, and viruses from water.

### Description of treatment units.

**Figure 1.**
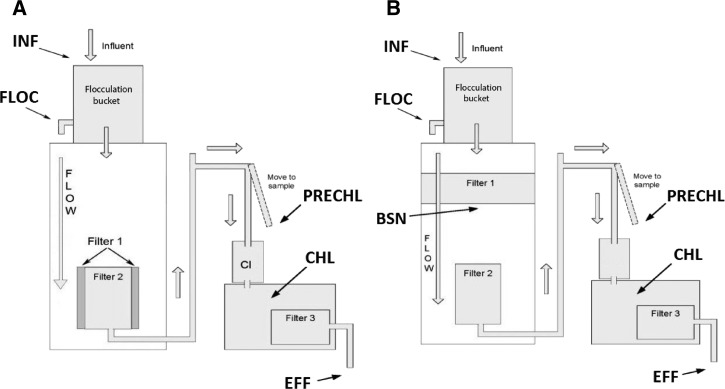
(**A**) Shows the schematic view for the sleeve unit design and (**B**) disk unit and description of sampling sites for the biofilter systems.

The two biofilter systems were built by Amway, Ada, MI. They were designed with a foam material configured in a layer to provide mechanical filtration and a platform for biofilm formation, followed by a carbon filter block. In the biofilter sleeve unit (Figure 1A), the foam material was positioned vertically around the carbon filter and in the biofilter disk unit (Figure 1B), it was positioned horizontal to the carbon filter block. The biofilter systems are gravity driven, and the source water was poured into the first container (flocculation bucket) where it underwent flocculation prior to filtration to reduce turbidity and remove large particles. Water within flocculation bucket was treated with coagulants (aluminum sulfate and polyaluminum chloride [PAC]) and allowed to settle for 1 hour on non-challenge days, and until turbidity measured below 3 nephelometric turbidity units (NTU), and a minimum of 2 hours, on challenge days. After flocculation, the water moved into the second container where it passed through the foam filter and then passed through the carbon filter block. The water then passed over a chlorine tablet and into a holding reservoir. The chlorine concentration in the lower bucket was controlled by flow rate, and a patented design (U.S. Patent No. D634397) that provides a blend of chlorinated and non-chlorinated water. The lower bucket served a dual purpose of extending contact time with chlorine as well as providing safe storage of treated water. This means that holding times within the chlorine stage within the lower bucket were dependent on the user. Chlorinated water in the final effluent flowed from the system via a tap, where the water passed through a second carbon filter for dechlorination to levels suitable for consumption. Reduction of chlorine levels also serve to provide water without a strong chlorine taste that can be repellant to some users.

### Test water composition.

Two types of water (maintenance and challenge water) were used for the evaluation of the biofiltration units per NSF Protocol P231 for testing of microbiological water purifiers (Table 1). The maintenance water was used for the development of the biofilm on the surface of the foam filter and to maintain the system between challenges. Total organic carbon (TOC) levels and turbidity parameters were obtained by adding test dust and humic acid. Sodium hydroxide and hydrochloric acid were used to adjust pH. Water samples were analyzed for TOC at the Amway Corporation Laboratories.

On test days, the challenge water was seeded with representative organisms for enteric virus, bacteria, and parasites. Bacteriophages MS2 and P22 served as surrogate organisms for enteric viruses, *Raoultella terrigena*, represented enteric bacteria, and heat-killed *Cryptosporidium parvum* oocysts served as parasitic protozoan pathogens with influent levels between 9.98 × 10^4^ ± 1.26 × 10^4^ PFU/mL, (*N* = 24); 4.63 × 10^6^ ± 7.59 × 10^5^ PFU/mL, (*N* = 22); 7.81 × 10^8^ ± 7.59 × 10^7^ CFU/100 mL (*N* = 26); and 1.23 × 10^7^ ± 3.24 × 10^6^ oocysts/L (*N* = 8), respectively (Figure 1).

### Microbial preparation and enumeration.

Bacteriophage MS2 (ATCC 15597-B1) was prepared using a double agar overlay and assayed in accordance with a modified version of Environmental Protection Agency (EPA) Method 1602 without MgCl_2_.^18^ In this method, a 2 mL sample volume of appropriate dilution was mixed with 2.5 mL molten trypticase soy agar (Trypticase^™^ soy agar; Beckton, Dickenson, and Company, Sparks, MD) and 0.5 mL log-phase host bacteria *Escherichia coli* (ATCC# 700891), and then poured into a petri plate containing a bottom layer of trypticase soy agar medium. After solidification, the plates were incubated at 36 ± 1.0°C for 16–24 hours prior to enumeration of plaques. Plaques were counted, and the MS2 concentration was computed as plaque-forming units (PFU) per milliliter (mL). Samples were analyzed over wide range of concentrations in quadruplicate. MS2 was added to the challenge water to a targeted concentration of > 1 × 10^5^ PFU/mL.

Bacteriophage P22 were prepared and assessed with a similar modified EPA Method 1602.^18^ For this phage, *Salmonella typhimurium* (HER #1023, Félix d'Hérelle Reference Center for bacterial viruses) was used as a host.

*R. terrigena* (ATCC 33257) was assayed using membrane filtration technique according to American Public Health Association (APHA) standard method 9222 B.^19^ Filtered samples were grown on mENDO media (mENDO agar LES; Beckton, Dickenson, and Company). Samples were incubated for 24 ± 2 hours at 35 ± 2°C prior to enumeration. *R. terrigena* concentration was computed as colony-forming units (CFU) per 100 mL of water. Live *R. terrigena* was added to challenge water to target concentrations of 1 × 10^6^ CFU/100 mL.

Heat-inactivated *C. parvum* oocysts obtained from Waterborne Inc. (New Orleans, LA) were included at target concentrations of 1 × 10^6^ oocysts/L. For *C. parvum* analysis, samples were analyzed using EPA method 1623 for *Cryptosporidium* and *Giardia* in water by filtration, immunomagnetic separation, and fluorescent-labeled antibodies.^20^ Influent and post-flocculation samples (INF and FLOC) were applied to slides, treated with immunofluorescent antibody stain and 4′,6-diamidino-2-phenylindole (DAPI) (EasyStain, Biotechnology Frontiers, Australia), and enumerated by fluorescence and differential interference contrast (DIC) microscopic examination. Samples collected from the basin (BSN) and prechlorinated (PRECHL) collection sites were filtered (Envirochek HV; Pall Corporation, Ann Arbor, MI), and materials retained on the filter were eluted with elution buffer and pelleted by centrifugation. The oocysts and cysts were magnetized by attachment of magnetic beads conjugated to anti-*Cryptosporidium* and anti-*Giardia* antibodies, separated from the extraneous materials using a magnet, and then oocysts and cysts were removed from the magnetic bead complex and enumerated as described previously.^20^

Biofilm maturation was determined and monitored using heterotrophic plate count (HPC) as outlined in APHA standard method 9215B.^19^ Water samples were taken from influent, flocculation, and prechlorination steps in both units; in addition, a sample was taken from the BSN site in the disk unit. These samples were filtered through sterile, hydrophilic mixed cellulose ester membranes (GN-6 Metricel^®^ S-Pack; Pall Corporation), and the filtered bacteria was grown on HPC media (mHPC; Beckton, Dickenson, and Company). Samples were incubated for 48 ± 2 hours at 35 ± 2°C, and the colonies were enumerated visually.

### Experimental protocol for biofilter evaluation.

The challenge water was seeded with representative organisms for enteric virus, bacteria, and parasite groups. Table 2 summarizes the microbial concentrations that were established as part of the experimental protocols.

The biofilter systems were tested in two phases: pre-conditioning phase and challenge phase. During the pre-conditioning phase, maintenance water was passed through the biofilter units for 24 days to establish a biofilm on the foam filters. Biofilm maturation was determined and monitored using HPC bacteria enumeration. The biofilm was considered mature when HPC levels plateaued. The challenge phase consisted of a weekly schedule in which 20 L/d of maintenance water was passed through the filter systems for 6 days, and then on the seventh day (a challenge day) challenge water seeded with the representative microorganisms was passed through the filter systems. To evaluate the contribution of the biofilm formation on performance of the biofilter units, an additional challenge was conducted after the filters were cleaned and the biofilm removed.

The systems were run a total of 113 days, with 1,696 L through each unit. A total of 11 challenge days were performed. The biofilm required 24 days to develop and mature by running general test water.

On challenge days, water samples (1 L) were taken from six biofilter system sites (Figure 1): influent (INF), flocculation (FLOC), basin (BSN), prechlorination (PRECHL), chlorination (CHL), and post-chlorination effluent (EFF). A total of five samples were collected from the sleeve unit and six from the disk unit each challenge day. To challenge the biofilter systems, 15-L carboys were filled with challenge water, seeded with the select microorganisms, mixed thoroughly, and 1 L aliquots were reserved as influent samples. The contents of the carboys were then transferred to the flocculation bucket of each unit. The coagulants were then added: first, aluminum sulfate (1.75 g) was added to the flocculation bucket and stirred in for 1 minute, then PAC (0.8 g) was added and stirred for another minute. The flocculation stage was considered complete when the turbidity measured 3 NTUs or less at the surface, after which samples were taken from the flocculation site. The flocculation valve was then opened, passing the water into the next chamber of the system. A 1 L sample was taken from the BSN site of the disk unit. Following the primary filtration step, 1 L samples were collected from the prechlorination location. The remainder of the water continued to pass into the chlorination reservoir. The next set of samples were collected from inside the chlorination reservoir (sodium thiosulfate was added to these samples to neutralize the chlorine). Finally, samples were collected at the effluent location.

### Experimental protocol for biofilm evaluation.

Prior to testing the contribution of biofilm formation on microorganism removal by the biofilter units, the foam filter in the disk unit was replaced with a new, unused disk filter as it was not easy to remove and wash the buildup of the biofilm after use. The foam in the sleeve unit was easily removed, so it was washed in dechlorinated water to remove the biofilm, and reused. To simplify the comparison, the flocculation bucket and chlorination step were removed from the biofilter systems. The challenge was then conducted without preconditioning (i.e., without biofilm).

Samples were collected from influent location (INF) prior to adding the challenge water, seeded with the select microorganisms, to the units. Samples were collected from the prechlorination location for the sleeve unit, and the prechlorination and basin sites for the disk unit. To generate a valid comparison between the systems without preconditioning, 2 L samples were taken at 50% and 120% of each units standing water volumes, designated V1 and V2, respectively (Table 3). The standing water volume, a characteristic of gravity-driven systems, is the amount of water remaining in the system once the influent water reaches equilibrium with the system outlet. 50% standing volume corresponds to 13 L in the sleeve unit and 17 L in the disk unit. 120% standing volume corresponds to 30 L in the sleeve unit and 41 L in the disk unit. These collection points were selected to detect changes in microbe removal as increasing volumes of water are passed through the system. A total of 680 L of seeded challenge water was passed through each of the filter systems. Samples were examined for concentration of bacteriophages MS2 and PS2, *R. terrigena,* and *C. parvum*, as described previously.

### Statistical analysis.

The goals of the statistical analysis were to examine the data for significant differences in reductions between systems, sampling sites, pre- and post-maturation conditions, and microorganism types. Data sets were assessed for normality using the Shapiro–Wilk test. Several data sets did not follow normal distribution, and no mathematical transformation of the data sets was found that produced normal distributions from all of the data sets. Therefore, nonparametric statistical tests were used for analysis. Analysis of variance (ANOVA) on ranks was used to detect statistical differences in ALR between sites in individual units. ANOVA on ranks was also used to compare the ALR between influent and flocculation, influent and prechlorination, flocculation and prechlorination, and prechlorination and chlorination for all organisms to examine potential differences between the sleeve unit and the disk unit. It was also used to compare the ALR between the sleeve unit and the disk unit between chlorination and effluent for MS2, P22, and *R. terrigena* and between prechlorination and effluent sites for *C. parvum.*.

## Results

### Flow rates.

The observed flow rates in the sleeve unit averaged 371 ± 32.11 mL/min during challenge events and 386 ± 38.95 mL/min during non-challenge events. The disk unit experienced flow rates of 420 ± 37.19 mL/min during challenge events and 443 ± 72.01 mL/min during non-challenge events. During the experiments without biofilm development, the filters were cleaned and flow rates were increased to 820 mL/min and 740 mL/min for the sleeve and disk units, respectively.

### Evaluation of biofilter units with biofilm development.

Table 4 shows the ALR achieved by the sleeve and disk units over the course of 11 challenges. In both the sleeve and disk unit, there was statistically significant reduction of all organisms across sampling points (influent, flocculation, basin, prechlorination, chlorination, and effluent) (*P* ≤ 0.01). However, no statistically significant differences between the sleeve and disk were found when comparing each organism's ALR from the following intervals: flocculation (INF-FLOC), influent to prechlorination (INF-PRECHL), flocculation to prechlorination (FLOC-PRECHL), chlorination (PRECHL-CHL), chlorination to effluent (CHL-EFF) (MS2 virus, P22 virus, *R. terrigena*), or prechlorination to effluent (PRECHL-EFF) (*C. parvum*). The *P* value for the interval comparisons was ≤ 0.01. There was no significant difference between the overall performance (influent to effluent) of the sleeve unit and the disk unit (*P* ≤ 0.01). An alpha of 0.05 was used for statistical significance in all tests.

#### P22.

In the sleeve unit, P22 virus showed an overall 6.16 ± 0.64 ALR from influent to effluent over 11 challenges. The foam, carbon stage, and biofilm accounted for 4.50 ± 0.42 ALR alone (FLOC-PRECHL). Chlorination (PRECHL-EFF) could not be fully evaluated due to low incoming concentration; it accounted for a measurable 0.16 ± 0.31 ALR. If tested in isolation from the rest of the system with a high concentration of challenge organisms, a higher ALR would be expected between the prechlorination and effluent sampling points. On the basis of the chlorine residual and contact times tested in isolation from the rest of the system with a high concentration of challenge organisms, a higher ALR would be expected between the prechlorination and effluent sampling points.

In the disk unit, P22 showed an overall 6.24 ± 0.46 ALR (influent to effluent) over 10 challenges. The carbon filter showed 2.40 ± 0.04 ALR (basin to prechlorination). The contribution of the carbon filter in contrast to the foam filter and biofilm could only be evaluated in the disk system as there was no way to sample these separately in the sleeve unit. Prechlorination to effluent accounted for > 0.01 ± 0.00 ALR in the disk unit (see Table 4).

#### MS2.

With biofilm maturation, the sleeve unit exhibited a total removal and inactivation of 4.55 ± 1.26 ALR across the entire system in nine challenges (influent to effluent). The combined foam, carbon filter, and biofilm stage accounted for 2.92 ± 0.51 ALR (flocculation to prechlorination). The chlorination step (prechlorination to effluent) in the sleeve unit inactivated the remaining MS2 virus. From the prechlorination step to the chlorination basin > 0.03 ± 0.10 ALR were observable (see Table 4).

In the disk unit, MS2 showed an overall 4.55 ± 1.04 ALR (influent to effluent). Of this, 1.78 ± 1.30 ALR was from the flocculation stage (influent to flocculation). The carbon filter showed 0.54 ± 0.50 ALR (basin to prechlorination). The contribution of the carbon filter in contrast to the foam filter and biofilm could only be evaluated in the disk system as there was no way to sample these separately in the sleeve unit.

#### R. terrigena.

In the sleeve unit, *R. terrigena* bacterium showed an overall 8.47 ± 0.55 ALR over 10 challenges (influent to effluent). Foam, carbon filtration, and biofilm (flocculation to prechlorination) accounted for 5.82 ± 1.09 ALR. Chlorination (PRECHL-EFF) showed > 1.72 ± 1.01 ALR.

In the disk unit, *R. terrigena* was sampled in 9 to 11 challenges (see Table 4). It showed an overall 8.36 ± 0.69 ALR (INF-EFF) over the challenges (*N* = 9). The foam and biofilm layer alone (FLOC-BSN) accounted for 2.74 ± 1.30 ALR, and the carbon filter (BSN-PRECHL) was responsible for 3.98 ± 1.49 ALR (*N* = 10). The contribution of the carbon filter in contrast to the foam filter and biofilm could only be evaluated in the disk system as there was no way to sample these separately in the sleeve unit. Chlorination (PRECHL-EFF) process accounted for > 0.73 ± 0.58 log_10_ reduction in the disk unit (*N* = 10).

#### C. parvum.

This pathogen was sampled in three to four challenges. The sleeve unit had a total of 6.46 ± 0.64 ALR over the entire system (INF-EFF). In the foam and carbon layer (FLOC-PRECHL), 5.51 ± 0.85 ALR was observed. The effect of the chlorination process on *C. parvum* was not quantified.

In the disk unit, *C. parvum* showed a total of 6.45 ± 0.86 ALR across the whole system (INF-EFF). The foam/biofilm layer by itself (FLOC-BSN) removed 3.71 ± 0.51 ALR of *C. parvum* and the carbon filter (BSN-PRECHL) removed 1.98 ± 0.89 ALR. The contribution of the carbon filter in contrast to the foam filter and biofilm could only be evaluated in the disk system as there was no way to sample these separately in the sleeve unit. The effect of the chlorination process on *C. parvum* reduction was not quantified.

### Chlorine stage.

The free chlorine measured an average of 2.14 ± 1.76 mg/L in the sleeve unit and 3.33 ± 1.85 mg/L in the disk unit as the water flowed over a chlorine tablet and into the reservoir. Contact time of chlorine within the reservoir was less than 10 minutes during challenges. Final effluent (after dechlorination) measured an average of 0.00 ± 0.00 mg/L in both units.

## Exploration of microbial reductions by biofilter units without biofilms.

A preliminary investigation into the removals associated with the foam biofilter units without a biofilm was undertaken. Table 5 displays the log_10_ reductions recorded during a challenge event. The sleeve unit had two sampling points in this challenge (equivalent to influent and prechlorination in the previous studies). The disk unit had an additional sample collection point that allowed the removal capacity of the isolated foam disk to be examined. This data represent the systems challenged without biofilm conditioning. The designation PRECHL is used for consistency, but as there were no chlorination steps involved in this challenge, the PRECHL point should be considered the endpoint for the entire system of this experiment.

### MS2.

The sleeve unit exhibited a total of 3.5 ALR of MS2 overall (INF-PRECHL) averaged over the two volume points. The disk unit showed 0.3 ALR across the foam layer without biofilm (INF-BSN); 3.5 ALR across the entire system through two time points (INF-PRECHL).

### P22.

The sleeve unit exhibited 3 ALR in total (INF-PRECHL) over two volume points. The disk unit showed 3 ALR overall (INF-PRECHL) across two volume points. The foam layer without biofilm (INF-BSN) contributed 0.4 ALR over two volume points. The sleeve unit experienced 4 ALR in total (INF-PRECHL) over the two volume points. The disk unit had 4 ALR overall (INF-PRECHL) over the two volume points. The foam layer of the disk unit had 4.95 ALR (INF-BSN), though this result is influenced by the limit of detection.

### *C. parvum*.

The sleeve unit showed a 3.5 ALR in total (INF-PRECHL) over the volume points. The disk unit experienced a 4 ALR overall (INF-PRECHL). The foam layer without biofilm contributed 0.2 ALR (INF-BSN).

## Discussion

The overall goal of this study was to determine the performance of the two multi-barrier filtration systems that used a foam material in a sleeve and disk format as a filter and to support a biofilm. This assessment focused on performance differences between the two configurations.

In both the sleeve and disk units, there was a significant difference between the initial pathogen concentrations at the influent and at the effluent (*P* ≤ 0.01). There was no significant difference between the overall performance of the sleeve unit and the disk unit (*P* ≤ 0.01) during any stage of the system (flocculation, filtration, or chlorination). The alpha value used for significance for all tests was 0.05.

In both the sleeve unit and the disk unit, the organisms were reduced to non-detectable levels. Within the units, the majority of reduction occurred between flocculation and the pre-chlorine stage (after the carbon filter), showing that the foam and carbon filtration system components were highly effective in pathogen removal. Within the disk unit, the effect of the foam/biofilm layer and the carbon filter were examined separately. The carbon filter demonstrated better capability to reduce *R. terrigena* and P22 than the foam (*P* ≤ 0.01). The foam/biofilm was better able to reduce MS2 and *C. parvum* compared with the carbon filter (*P* ≤ 0.01). It is not clear whether this might be due to attachment to the carbon or biofilms. Abbaszadegan and others examined a system with carbon filters and UV light disinfection. In their analysis, the carbon stage of the system contributed 2 log_10_ reduction of viruses.^21^ This is nearly equivalent to the observed log_10_ reduction values of P22 in the disk unit but greater than with the observed reduction of MS2 in the current study.^21^ Abbaszadegan and others also found that the carbon filter demonstrated up to 3 log_10_ removal of *C. parvum* in accordance with National Sanitation Foundation standards. This is 1 log_10_ higher than the observed reduction in the biofilter disk unit attributable to the carbon alone in the current study.^21^ The alpha value used for significance for all tests was 0.05.

Accurate quantification of the inactivation due to chlorination phase was complicated because of the low starting concentration of organisms in the water after treatment entering the disinfection chamber of the units. The chlorine stage reduction values were calculated to be no more than 1.72 ± 1.01 ALR, but the chlorine stage likely has additional reduction capacity that was not observable with the current study design. During challenges, free chlorine residuals ranged from 0.8 to up to 2.1 mg/L (2.14 ± 1.76 average) in sleeve unit prior to dechlorination and from 0.8 to 3.5 mg/L (3.33 ± 1.85 average) in the disk unit of these biofilter systems. Other point-of-use devices utilizing only chlorine demonstrated a baseline of 3 log_10_ reduction value.^6^ Devices incorporating coagulation along with chlorination also showed baseline log_10_ reduction values of 2–7 (varies by type: bacteria, virus, or protozoa).^6^ The Pur^®^ Water Purifier (Procter and Gamble, Cincinnati, OH) utilizing flocculation and chlorination demonstrated > 7.3 to > 9.96 log_10_ reduction for bacteria, ≥ 4.78 to > 6.06 log_10_ reduction for virus, and 3.55–4.32 for protozoa.^22^

The removal performance of the biofilter was explored without preconditioning the systems. This round of testing was meant to simulate the performance of new systems without a biofilm. This could happen when users neglect instructions and use the devices prior to the development of a mature biofilm. Two volumes were evaluated to examine how increase in water passed through the unit (representing two times the standing volume of the systems) influenced the removal.

It was anticipated that there would be less removal without preconditioning due to a lack of biofilm development. The preconditioning reduction values of MS2 virus, P22 virus, and *R. terrigena* at V1 in the sleeve unit were similar to post-conditioning reduction values. Thus, the role of the biofilm could not be shown except for the parasites, where preconditioning reduction values were lower for *C. parvum* by 1.81 ALR.

After V2 sampling in the sleeve unit, average bacteriophage MS2 reduction was approximately 1 log_10_ higher without a biofilm as compared with the filtration unit with mature biofilm formation. All other microbes showed average reduction values for the challenge organisms at least 2 log_10_ lower as compared with those seen from conditioned biofilter units. These results suggest that for systems without a biofilm, as a greater volume is pushed through the system there is generally a decrease in the removal of microorganisms, suggesting that the microbes initially attach to the filter and then detach as more water flows through the system. The presence of a biofilm may prevent the organisms from being released. There may be a limit as to how much microbe retention the biofilm can support; the release rate of pathogens, particularly protozoa, may be dependent on many variables.^12^

The data from the disk unit preconditioning data set show a notable decrease in the reduction values from flocculation to basin sampling locations compared with the post-conditioning data set, for all organisms except *R. terrigena*. FLOC to PRECHL pre-conditioning reduction values were at least 1 ALR lower than the post-conditioning reduction values.

**Figure 2.**
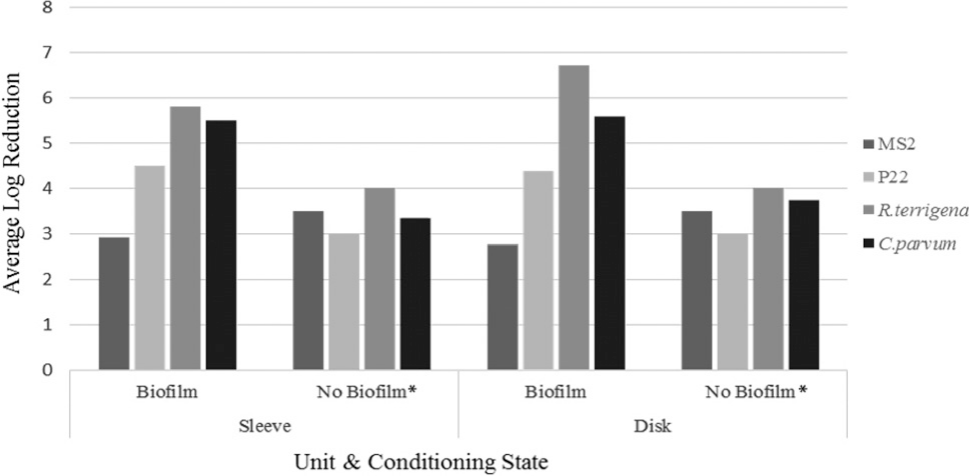
Displays the averaged V1 and V2 pre-biofilm reduction values in comparison to ALR for the post-biofilm values. * Results represent average of time point 1 and 2 reduction values.

These differences between the pre- and post-conditioning reduction values suggest that the biofilm layer itself may be contributing from 1 to 2 log_10_ reduction in the biofilter systems for P22 virus and *C. parvum* (see Figure 2). The role of the biofilm layer in MS2 and *R. terrigena* reduction is more ambiguous, with sleeve unit MS2 reductions and disk unit *R. terrigena* reductions demonstrating different patterns in reductions than the other challenge organisms. The causes of these differences should be investigated further, as should the role of biofilms in general. There was a difference in flow rates with and without biofilm development as well, without biofilms the flow rates averaged 449 and 320 mL/min more than when biofilms were present on the foam for the sleeve and disk units, respectively (comparison is using challenge event flow rate values). This could represent a key factor in the differences between the pre- and post-conditioning reduction values. Other studies using sand filtration and biofilms cite change in flow rates as contributing factors to changes in removal.^8^ In this study, the preconditioning flow rate was roughly two times the post-conditioning flow rate. Further investigation is needed to separate the effects and further understand the mechanisms of the biofilm and associated flow rates and hydraulic retention times impacting reduction efficiency.

In the sleeve unit, the removal performance improved for MS2 virus between V1 and V2 but decreased for all other challenge organisms. In the disk unit, removal performance for MS2 was more variable. MS2 virus and *R. terrigena* removal improved between V1 and V2 in the disk unit. Removal performance for P22 virus showed no change in the disk unit, but removal performance for *C. parvum* decreased. These varying results will require many more replicates and further experiments for investigating types of biofilms and the mechanisms involved in improving filtration.

A comparison of the sleeve and disk biofilter systems to other point-of-use filtration systems demonstrate removal and inactivation of microbial loads similar to, if not exceeding, those of other water filtration devices. In a review of point-of-use water purification devices, several different methods for removal and/or inactivation of microbial loads were compared: porous ceramic filtration (PCF), biosand filtration (BSF), polyethylene bottles with solar UV and heat disinfection (SODIS), free-chlorine treatment, and coagulation and chlorination treatment.^6^ When compared with ALR results calculated for the biofilter systems used in this study, it was found that the removal performance of sleeve and disk biofilter systems was similar to, if not exceeding, those in the review.^6^

### Practical considerations.

Apart from performance measures, practical factors such as ease of use must also be taken into account when considering the implementation of biofilter. In comparison to other gravity-fed point-of-use devices such as sand filters, the biofilter designs offer more portability. The major difference between the sleeve unit and the disk unit was the position of the foam filter. In practice, the foam disk configuration was more difficult to use. The foam disk was quite fragile and required greater care to not puncture the foam during the cleaning procedure. This fragility could lead to eventual damage resulting in the reduction in the ability of the system to remove pathogens, particularly *Cryptosporidium* that requires an intact physical barrier for removal, as it is resistant to inactivation by chlorine. The biofilm is disrupted when the foam layer of the biofilters are cleaned, which may produce variability in filtration performance.

The biofilter designs are multi-barrier systems and there are advantages and disadvantages to these designs. The foam system is light weight and has a small footprint compared with biosand filters. Initial setup of the biofilters was considerably less labor intensive and more straightforward. The biofilter materials also lend themselves a uniformity in construction that may be advantageous. The biofilter cartridge is vulnerable to mishandling when being installed. It must be screwed into a pipe located at the bottom of the basin, requiring a certain amount of torque that could easily be over-applied, or the cartridge could be incorrectly positioned.

Another possible weakness of the biofilter system is the need for additional substances to maintain full functionality, specifically the flocculation and chlorine steps. A constant supply of flocculating agents (aluminum sulfate and PAC) must be supplied to users of the biofilters to maximize settling efficiency. Overall system performance could become compromised in the event when the flocculating agents run out. A similar problem exists with the chlorine tablets used in the holding reservoir. In our evaluations, the chlorine tablets required replacement after 93 days of use, constituting 13–15 L water filtered each day. In the event when flocculation and chlorination substances are depleted and those substances become unavailable to users, the pathogen reduction power of the system may become compromised. According to the results of the post-flocculation and prechlorination removals in Table 4, the system would lose roughly 1–2 log_10_ reduction power over the entire system, though additional and more testing of this scenario may reveal a more definitive answer to this question.

In conclusion, these units meet the standards set forth by the U.S. EPA for microbial purifiers, 3 log_10_ reduction for protozoan cysts, 4 log_10_ for viruses, and 6 log_10_ reduction for bacteria.^23^ Although more in-depth performance evaluations should be conducted, the biofilter designs have demonstrated an ability to produce potable water. Performance under extreme conditions such as those produced from misuse, incorrect handling, and durability in the field would be beneficial to understanding how they will perform outside the laboratory.

### Future work.

Additional study should be conducted evaluating long-term use of the biofilter in a laboratory setting. This study should include long duration of use as well as large volumes and frequency of use to test the filter design under more strenuous conditions. Evaluation of filter performance before and after cleaning procedures should be performed more thoroughly to reinforce or refute the preliminary tests performed in this research study exploring the role of the biofilm in the biofilter system.

Additional work should evaluate the use of the filter in a field environment, monitoring important factors such as compliance, disease prevalence, and durability in a real-world setting. A performance test before and after the puncture of the foam disk or sleeve would provide for an understanding of how resilient the filter system would be to damage in the field. Evaluation of performance without the flocculation or chlorination steps would reveal a baseline standard of performance in the event of a disrupted supply chain for renewable materials.

## Figures and Tables

**Table 1:** Test water composition for challenging the biofilter units

Parameters	Maintenance water parameters	Challenge water parameters
Free chlorine	0.0 mg/L	0.0 mg/L
pH	6.5–8.5	9.0 ± 0.2
Total organic carbon (TOC)	2.48 ± 3.26 mg/L*	1.59 ± 1.14 mg/L*
Turbidity	30 NTU	≥ 30 NTU
Temperature	20 ± 5°C	4 ± 1°C

* Targeted TOC levels were 10 mg/L for maintenance water and > 10 mg/L for challenge water. However, levels plateaued on additional amounts of humic acid. This suggests that the humic acid used either had solubility issues with the organic fraction of the carbon, or a much higher percentage of inorganic carbon.

**Table 2:** Targeted and achieved organism concentrations for the biofilters' challenge events

Organism	Target influent concentration	Achieved average influent concentration
Bacteriophage MS2	1 × 10^5^ PFU/mL	9.98 × 10^4^ ± 1.26 × 10^4^ PFU/mL (*N* = 24)
Bacteriophage P22	1 × 10^5^ PFU/mL	4.63 × 10^6^ ± 7.59 × 10^5^ PFU/mL (*N* = 22)
*Raoultella terrigena*	1 × 10^6^ CFU/100 mL	7.81 × 10^8^ ± 7.59 × 10^7^ CFU/100 mL (*N* = 26)
*Cryptosporidium parvum* oocysts	1 × 10^6^ oocysts/L	1.23 × 10^7^ ± 3.24 × 10^6^ oocysts/L (*N* = 8)

**Table 3:** Sampling points as increasing volume was passed through filtration units (without biofilm formation)

Unit	50% Standing volume (V1)	120% Standing volume (V2)
Sleeve unit	13 L	30 L
Disk unit	17 L	41 L*

* After 41 L had passed the filter, the remaining volume flowing from the system was not sufficient to collect 2 L samples from basin and effluent. A 400 mL sample was collected at the effluent for bacterial analyses. 1 L from the standing volume was taken for *Cryptosporidium parvum* analysis rather than 2 L.

**Table 4:** Comparing log_10_ reduction averages of viruses, bacteria, and protozoa in two foam-based biofilter units after biofilm development

Sampling site	P22 *N* = 10*		MS2 *N* = 9		*R. terrigena N* = 10		*C. parvum N* = 4	
	Sleeve	Disk	Sleeve	Disk	Sleeve	Disk	Sleeve	Disk
INF-FLOC	1.50 ± 0.37†	1.60 ± 0.46	1.59 ± 1.09	1.78 ± 1.30	1.14 ± 0.36	1.14 ± 0.81∥	0.71 ± 0.32	0.83 ± 0.04
FLOC-BSN		2.22 ± 0.43		2.23 ± 0.69		2.74 ± 1.30		3.71 ± 0.51
BSN-PRECHL‡		2.40 ± 0.04		0.54 ± 0.50		3.98 ± 1.49		1.98 ± 0.89
PRECHL-EFF	> 0.16 ± 0.31	> 0.01 ± 0.00	0.03 ± 0.10	0.00 ± 0.00	> 1.72 ± 1.01	> 0.73 ± 0.58	> 0.00 ± 0.00§	> 0.00 ± 0.03§
FLOC-PRECHL	4.50 ± 0.42	4.38 ± 1.00	2.92 ± 0.51	2.77 ± 0.84	5.82 ± 1.09	6.72 ± 2.11	5.51 ± 0.85	5.60 ± 0.88§
INF-PRECHL	6.00 ± 0.49	6.23 ± 0.46	4.51 ± 1.25	4.55 ± 1.04	6.96 ± 1.34	7.77 ± 0.87	6.23 ± 0.70§	6.52 ± 0.71§
INF-EFF (Total)	≥ 6.16 ± 0.64	≥ 6.24 ± 0.46	≥ 4.55 ± 1.26	≥ 4.55 ± 1.04	≥ 8.47 ± 0.55	≥ 8.36 ± 0.69∥	≥ 6.46 ± 0.64§	≥ 6.45 ± 0.86§

* *N* = Number of events used in calculation, unless otherwise noted.

† ± = Standard deviation.

‡ After foam disc before carbon, this could only be evaluated for the disk configuration.

§ *N* = 3 for the evaluation of some *Cryptosporidium* reductions.

∥ 9 events used to calculate INF-FLOC and INF-EFF reductions.

**Table 5:** Microbial log_10_ reductions for INF-PRECHL stage without biofilm development

Challenge organism	Sleeve		Disk	
	V1	V2	V1	V2
MS2 virus	3	4	3	4
P22 virus	4	2	3	3
*R. terrigena*	5	3	3	5
*C. parvum*	4	3	4	4
